# Indomethacin attenuates mechanical allodynia during the organization but not the maintenance of the peripheral neuropathic pain induced by nervus ischiadicus chronic constriction injury

**DOI:** 10.1590/1414-431X20209255

**Published:** 2020-04-27

**Authors:** P. Medeiros, I.R. dos Santos, A.C. Medeiros, J.A. da Silva, S.H. Ferreira, R.L. de Freitas, N.C. Coimbra

**Affiliations:** 1 Laboratório de Neurociências da Dor & Emoções, Departamento de Cirurgia e Anatomia, Faculdade de Medicina de Ribeirão Preto, Universidade de São Paulo, Ribeirão Preto, SP, Brasil; 2 Centro Multiusuários de Neuroeletrofisiologia, Departamento de Cirurgia e Anatomia, Faculdade de Medicina de Ribeirão Preto, Universidade de São Paulo, Ribeirão Preto, SP, Brasil; 3 Laboratório de Neuroanatomia e Neuropsicobiologia, Departamento de Farmacologia, Faculdade de Medicina de Ribeirão Preto, Universidade de São Paulo, Ribeirão Preto, SP, Brasil; 4 Laboratório de Dor e Imflamação, Departamento de Farmacologia, Faculdade de Medicina de Ribeirão Preto, Universidade de São Paulo, Ribeirão Preto, SP, Brasil; 5 Departamento de Psicologia, Universidade Federal de Juiz de Fora, Juiz de Fora, MG, Brasil; 6 Instituto de Neurociências e Comportamento, Ribeirão Preto, SP, Brasil; 7 Instituto de Ciências Biomédicas, Universidade Federal de Alfenas, Alfenas, MG, Brasil

**Keywords:** Non-steroidal anti-inflammatory drugs, Indomethacin, Chronic constriction injury of the nervus ischiadicus, Neuropathic pain, Mechanical allodynia, von Frey test

## Abstract

The neurochemical mechanisms underlying neuropathic pain (NP) are related to peripheral and central sensitization caused by the release of inflammatory mediators in the peripheral damaged tissue and ectopic discharges from the injured nerve, leading to a hyperexcitable state of spinal dorsal horn neurons. The aim of this work was to clarify the role played by cyclooxygenase (COX) in the lesioned peripheral nerve in the development and maintenance of NP by evaluating at which moment the non-steroidal anti-inflammatory drug indomethacin, a non-selective COX inhibitor, attenuated mechanical allodynia after placing one loose ligature around the nervus ischiadicus, an adaptation of Bennett and Xie's model in rodents. NP was induced in male Wistar rats by subjecting them to chronic constriction injury (CCI) of the nervus ischiadicus, placing one loose ligature around the peripheral nerve, and a sham surgery (without CCI) was used as control. Indomethacin (2 mg/kg) or vehicle was intraperitoneally and acutely administered in each group of rats and at different time windows (1, 2, 4, 7, 14, 21, and 28 days) after the CCI or sham surgical procedures, followed by von Frey's test for 30 min. The data showed that indomethacin decreased the mechanical allodynia threshold of rats on the first, second, and fourth days after CCI (P<0.05). These findings suggested that inflammatory mechanisms are involved in the induction of NP and that COX-1 and COX-2 are involved in the induction but not in the maintenance of NP.

## Introduction

According to the International Association for the Study of Pain (IASP), pain is an unpleasant sensory and emotional experience associated with actual or potential tissue injury or described regarding such damage (1). Pain is a signal to prevent further lesion or to avoid tissue injury. The genesis and duration of chronic pain are critically different from those of acute pain, and the underlying mechanisms of chronic pain need to be more deeply studied (2).

Neuropathic pain (NP) is related to the reaction of nervous system tissue to neural damage. NP can be induced by lesions of the peripheral nervous system (PNS) caused by tumor invasion, metabolic diseases, infection, neurotoxic chemicals, and mechanical trauma leading to pathophysiological changes in either the PNS or the central nervous system (CNS) (3).

The incidence of chronic pain in the world is approximately 8% (4–5). The prevalence of NP is estimated to be 23% (5). NP is an unpleasant and debilitating condition that causes a severe impact on the quality of life of patients (6). The management of patients affected by NP is complicated, involving a multidisciplinary team for alleviating symptoms and available treatments are frequently ineffective.

Thus far, several models of peripheral NP have been developed in rodents. These experimental neurological procedures aim to mimic the clinical symptoms reported in humans after central or peripheral nerve injury, diabetic neuropathy, chemotherapy, and inflammation. The majority of NP models in rodents are based on the compression or section of the targeted nerve (7).

The nervus ischiadicus chronic constriction injury (CCI) model, as proposed by Bennett et al. (8), is an animal model of peripheral neuropathy. We have recently reported an improvement in this procedure in a laboratory animal model of NP, inducing CCI of the right nervus ischiadicus through a single loose ligature in an adaptation of the NP model described by Bennett and Xie in 1988 (9–11). The modified model produced sensory, affective, and cognitive disorders in rodents (9–12). In addition, mechanical allodynia has been recorded twenty-one days after nervus ischiadicus CCI surgery (10,11).

In fact, NP manifests itself through neurochemical changes in both the PNS and the CNS, affecting the peripheral nerve, spinal, and cortical structures (13). In the periphery, neuropathic lesions trigger peripheral sensitization that induces an inflammatory process and abnormal long-term neural activity along the primary afferent pathways (14).

Non-steroidal anti-inflammatory drugs (NSAIDs) are used to treat pain and reduce fever, and they have anti-inflammatory properties (15). Non-selective and selective cyclooxygenase (COX) inhibitors are partially effective in reducing NP symptoms induced by partial transection of the nervus ischiadicus (16), indicating that pro-inflammatory prostaglandins are involved in NP. In the present study, we evaluated whether indomethacin, an NSAID that inhibits the cyclooxygenase 1 and 2 (COX-1 and COX-2) enzymes, can attenuate mechanical allodynia in an adapted CCI model of NP generated by placing a single loose ligature around a peripheral nerve.

## Material and Methods

### Animals and housing conditions

Adult male Wistar rats (n=208), weighing 250–300 g, from the animal care facility of the University of São Paulo (USP; Campus of Ribeirão Preto) were used. Animals were housed in groups of four with free access to food and water. The housing room was kept at constant room temperature (22±1°C) and illuminated on a fixed light-dark cycle (lights on 07:00–19:00 h). All protocols were in compliance with the recommendations of the Committee for Ethics in Animal Experimentation of the Ribeirão Preto Medical School of the University of São Paulo (Processes 015/2005 and 036/2017), which agree with the animal research ethics adopted by the National Council for Animal Experimentation Control and with the IASP guidelines for pain research on animals (17). Each animal was used in a single experimental group and all efforts were made to minimize their discomfort.

### Neuropathic pain induction

An adapted model of chronic constriction injury (CCI) of the nervus ischiadicus was used to induce NP. A single ligature consisting of a chromic catgut suture (Bioline Fios Cirúrgicos Ltda., Brazil) was loosely placed around the right nervus ischiadicus (9–12). Each animal, in turn, was anesthetized with a mixture of ketamine (União Química Farmacêutica Nacional, Brazil) and xylazine (Hertape/Calier, Brazil) (92 mg/kg and 9.2 mg/kg, respectively, *ip*). The right hind paw was shaved and moistened with a disinfectant, and the common nervus ischiadicus was exposed at the mid-thigh level by blunt dissection through the biceps femoris. Then, proximal to the nervus ischiadicus trifurcation, approximately 7 mm of the nerve was freed of adhering tissues, and one loose ligature (4.0 chromic gut) was placed around the peripheral nerve. The wound was then closed by suturing the muscle with chromic catgut in a continuous suture pattern. Finally, the skin was closed with 3-0 black braided silk sutures (Teleflex Medical OEM, USA) in a horizontal mattress suture pattern. Sham surgery was performed by exposing the nervus ischiadicus as described above, without ligation. The animals were then transferred to their home cages and left to recover.

### Measurement of mechanical allodynia

Mechanical allodynia was assessed by von Frey test filaments (Stoelting, USA). Each animal was placed in a transparent acrylic cage (22×16.5×14 cm) with a wire-grid floor for approximately 20 min to allow behavioral acclimation to the novel environment before testing. von Frey's filaments were then applied through the mesh floor in ascending order from 10 to 100 g to the plantar surface at the center of the paw or the base of the third or fourth toe of the injured and uninjured hind paw for approximately 3–4 s per filament to induce the withdrawal reflex. The time interval before the application of the next filament was at least 10 s. The data are reported as mechanical withdrawal thresholds (MWTs) in grams. The MWT was evaluated before and after CCI or sham surgery and before and after pharmacological treatments. The test was repeated three times for both hind paws (ipsilateral and contralateral paws were tested in alternation) with an interval of at least 2 min between measurements. Before surgery, it was confirmed that there was no difference in the basal responses of the right and left paws (18).

### Systemic drug administration: effect of indomethacin on mechanical allodynia

**Figure 1:**
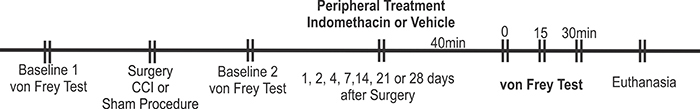
Timeline of the experimental procedure. The animals (n=6 to 9 per group) were divided into four groups: vehicle/sham, vehicle/chronic constriction injury (CCI), indomethacin/sham, and indomethacin/CCI. The mechanical-stimulus-induced response threshold was measured once before the CCI or sham procedures (day 0) and once 1, 2, 4, 7, 14, 21, or 28 days after surgery. Pretreatment with indomethacin (2 mg/kg) or vehicle was performed intraperitoneally, and indomethacin or vehicle was acutely administered in each group of rats. Mechanical allodynia was measured at 0, 15, and 30 min after the intraperitoneal drug or vehicle administration. The time point of 0 min indicates the end of the 40-min interval after the intraperitoneal administration of drugs. Subsequently, the animals were euthanized.

To evaluate an NSAID that inhibits COX-1 and COX-2 during NP, indomethacin (2 mg/kg) (19) (or vehicle) was intraperitoneally and acutely administered in each group of rats at different time windows (1, 2, 4, 7, 14, 21, and 28 days) after CCI or sham surgical procedures (day 0). Mechanical allodynia was measured at 0, 15, and 30 min after the intraperitoneal drug or vehicle administration. The time point of 0 min marked the end of the 40-min interval after the intraperitoneal administration of drugs (Figure 1) (20,21).

The animals were subjected to the experimental tests in the light phase of the light-dark cycle, and the treatment was always performed at the same time of the day and by the same researcher. The sessions were performed by a trained researcher who analyzed the experimental data in a blinded manner. After the end of the experimental test, the animals were euthanized with ketamine (92 mg/kg) and xylazine (9.2 mg/kg).

### Drugs

1-(4-Chlorobenzoyl)-5-methoxy-2-methyl-3-indoleacetic acid (indomethacin) (2 mg/kg) (Sigma-Aldrich Brazil, Brazil) was diluted in Tris/HCl (2-amino-2-(hydroxymethyl) propane-1,3-diol/hydrochloric acid) buffer, pH 8.0.

### Statistical analysis

Data are reported as means±SE. The mechanical allodynia results from the von Frey test were analyzed by a repeated-measures two-way analysis of variance (ANOVA). In the case of a significant treatment-by-time interaction, one-way ANOVAs were performed, followed by Duncan's *post hoc* test at each time interval. P<0.05 was considered statistically significant. The software used for statistical analyses was SPSS 13 (IBM, USA), and the graphs were plotted in GraphPad Prism version 7.0 (USA).

## Results

### Effect of indomethacin on mechanical allodynia

Animals of CCI group showed mechanical allodynia at 1, 2, 4, 7, 14, 21, and 28 days after CCI. The systemic administration of indomethacin attenuated mechanical allodynia at 1, 2, and 4 days after CCI induction. No change was observed in the MWT in the contralateral paw of the CCI or sham procedure (not shown).

**Figure 2:**
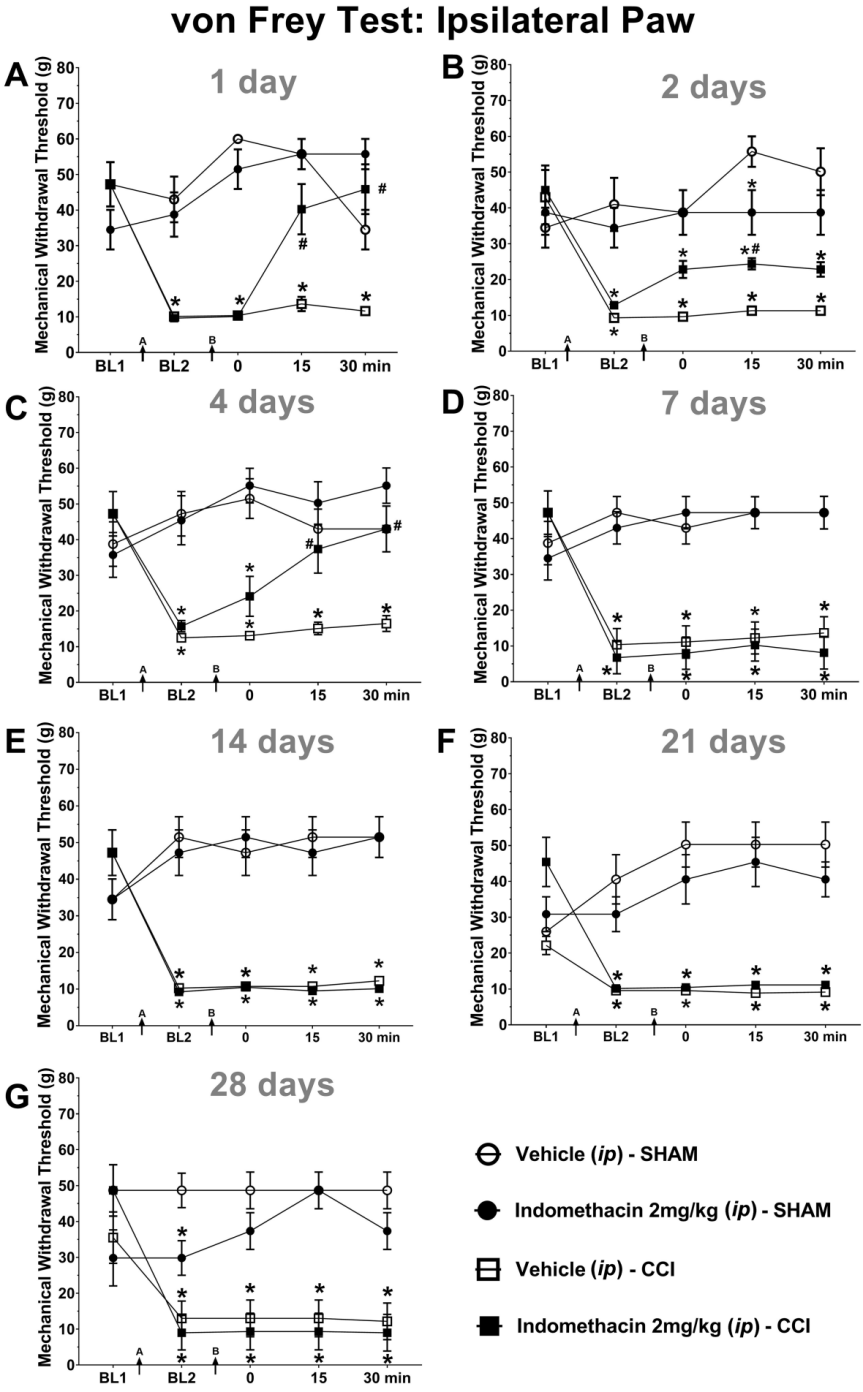
**A** to **G**, Effect of the intraperitoneal (*ip*) administration of indomethacin on the genesis and maintenance of neuropathic pain induced by an adapted model of chronic constriction injury (CCI) of the nervus ischiadicus in the right hind paw of Wistar rats. BL1: von Frey's test baseline was recorded before each procedure. Arrow A: Nervus ischiadicus CCI or sham procedure. BL2: New baseline recorded at 1, 2, 4, 7, 14, 21, and 28 days after the sham or CCI procedure. Arrow B: Administration of indomethacin or vehicle in rats subjected to sham or CCI procedures (von Frey's test applied to the ipsilateral paw until 30 min after injection). The data are reported as means±SE. *P<0.05, compared with the sham-vehicle treated group; ^#^P<0.05 compared with the CCI-vehicle-treated group (repeated-measures two-way ANOVA followed by Duncan's *post hoc* test).

At day 1 after CCI, there were statistically significant effects of treatment [F_(3,28)_=18.89; P<0.0001] and of time [F_(4,25)_=15.53; P<0.0001] on the MWT, as well as a significant treatment-by-time interaction [F_(12,71)_=11.43; P<0.0001]. There was a significant decrease in mechanical allodynia in rats after indomethacin administration compared with vehicle administration at the 15- and 30-min time points (P<0.05), according to Duncan's *post hoc* test (Figure 2A).

At day 2 after CCI induction, there were statistically significant effects of treatment [F_(3,25)_=12.55; P<0.0001] and of time [(F_(4,22)_=5.70; P<0.01] on the MWT, as well as a significant treatment-by-time interaction [F_(12,62)_=3.26; P<0.01], according to the repeated-measures two-way ANOVA. Treatment with indomethacin significantly attenuated mechanical allodynia (P<0.05, Duncan's *post hoc* test) at 15 min after administration in CCI rats. Indomethacin also increased the MWT at 15 min after administration in the sham group compared with the vehicle-treated sham group (Figure 2B), and this effect was statistically significant.

At day 4, there were statistically significant effects of treatment [F_(3,27)_=16.43; P<0.0001] and of time [F_(4,24)_=3.71; P<0.01] on the MWT, as well as a significant treatment-by-time interaction [F_(12,68)_=4.63; P<0.0001]. Indomethacin decreased mechanical allodynia at 15 and 30 min after administration (Duncan's *post hoc* test; P<0.05) (Figure 2C).

At 7 days after CCI, analysis of the MWT showed significant effects of treatment [F_(3,28)_=54,97; P<0.0001] and of time [F_(4,25)_=4.25; P<0.001], as well as a significant treatment-by-time interaction [F_(12,71)_=4.28; P<0.0001]; at 14 days after CCI, analysis showed significant effects of treatment [F_(3,28)_=27.56; P<0.0001] and of time [F_(4,25)_=4.02; P<0.01], as well as a significant treatment-by-time interaction [F_(12,71)_=7.09; P<0.0001]; at 21 days after CCI, there were significant effects of treatment [F_(3,24)_=24.72; P<0.0001] and of time [F_(4,21)_=3.28; P<0.05], as well as a significant treatment-by-time interaction [F_(12,59)_=7.05; P<0.0001] according to the repeated-measures two-way ANOVA followed by Duncan's *post hoc* test. Indomethacin did not modify the MWT in sham or CCI rats at days 7, 14, or 21 after surgery (Figure 2D to F).

At 28 days after CCI, there were significant effects of treatment [F_(3,20)_=10.81; P<0.0001] and of time [F_(4,17)_=10.86; P<0.0001] on the MWT, as well as a significant treatment-by-time interaction [F_(12,47)_=4.21; P<0.0001], according to the repeated-measures two-way ANOVA. The postsurgical baseline MWT values of sham rats treated with vehicle were significantly different from those of sham rats treated with indomethacin (2 mg/kg, *ip*) (Duncan's *post hoc* test, P<0.05) (Figure 2G).

The data of the effects of indomethacin (2 mg/kg, *ip*) on mechanical allodynia are reported in Supplementary Table S1.

## Discussion

In this study, the Wistar rats subjected to the adapted model of CCI using only one ligature around the nervus ischiadicus showed mechanical allodynia. The NSAID indomethacin, a non-selective COX inhibitor, attenuated mechanical allodynia during the first stage of NP development (from the first to the fourth day after the CCI procedure).

In fact, the pharmacological treatments of pain, including pregabalin (a lipophilic GABA analogue with a substitution at the 3'-position to facilitate diffusion across the blood-brain barrier), gabapentin (another analogue of GABA), duloxetine (a serotonin and noradrenaline reuptake inhibitor), and various tricyclic antidepressants, have strong recommendations for use and are recommended as first-line treatments for peripheral and central neuropathic pain (22,23). Such approaches are usually associated to NSAIDs (24) and opioid analgesics. Indomethacin is a synthetic NSAID introduced in the early 1960s (25) and was initially utilized for the treatment of pain and inflammation from rheumatological diseases. Inhibiting the release and biosynthesis of prostaglandins, indomethacin has antipyretic, analgesic, and anti-inflammatory properties (19,26–28). In addition, indomethacin acts through the inhibition of the COX enzymes, consisting of an important therapeutic intervention for the management of acute and chronic pain (29).

The pharmacological target of NSAIDs are the enzymes that catalyze the conversion of arachidonic acid in prostaglandins (PGs), which are the main mediators of exaggerated pain sensation (29–30). Therefore, PGs may play an important role in the induction of NP. NSAIDs are strong COX inhibitors that are used in the management of chronic, inflammatory, and postoperative pain (31). However, they have serious side effects (gastro-intestinal complications and, at high levels, hepatotoxicity and nephrotoxicity) (32–33), which make them not very safe choices for the long-term management of chronic pain.

In the current study, indomethacin attenuated mechanical allodynia until the fourth day after CCI induction, whereas it proved to be ineffective from the seventh to the twenty-eighth days after CCI. Indeed, the treatment with indomethacin decreased the mechanical allodynia during the initial phase rather than during the chronic phase when that effect was not observed. These findings could be explained if we consider that the sensitization of supraspinal nociceptive transmission might be involved in the expression of chronic pain (34). Interestingly, indomethacin attenuated mechanical allodynia at 15 and 30 min after the application of mechanical stimuli. Intraperitoneal treatment with indomethacin caused a much higher effect at the first and fourth days compared to the second day after the peripheral nerve injury, possibly because of the biological variability of the laboratory animals. It is also possible that on the second day after CCI of the nervus ischiadicus, more pronounced inflammatory reactions can surpass the pharmacological effect of indomethacin. In fact, after the nerve injury, Schwann cells and resident immune cells such as mast cells and macrophages are the first to react against cellular injury.

Molecular signaling from damaged axons results in the activation of the extracellular stimulus-related (ERK) mitogen-activated protein (MAP) kinase signaling pathway in Schwann cells, as one of the earliest events triggering the release of inflammatory mediators and recruiting immune cells to the damaged nerve (35). Resident mast cells degranulate inflammatory mediators, including histamine, serotonin, nerve growth factor, and leukotrienes, which can sensitize nociceptors and also contribute to the recruitment of neutrophils, the first cells to infiltrate damaged tissue. Neutrophil infiltration to the site of injury is acute, peaking within the first few hours after injury and declining after 3 days, with levels remaining elevated (36). Then, that phenomenon could explain the fact that on the second day after the injury the IP-treatment with indomethacin caused lower effects.

Peripheral nerve damage can result in chronic NP in multiple ways (37). While the insult may be localized in a specific division of either the PNS or the CNS, the pathophysiological response that leads to chronic pain is diffuse. Peripheral terminals of pain-processing unmyelinated C-fibers and thinly myelinated Aδ fibers can spur the development of NP after being affected by metabolic damage, toxins, medications, cytokines, and other inflammatory mediators (38), resulting in fiber density changes and neuronal hyper-excitability (39).

For example, a short-lived inflammation was induced in a rat hind paw by intradermal injection of a very low dose of the inflammogen carrageenan. The resultant inflammation (localized redness with minimal swelling) was associated with acute hyperalgesia, detected as a decrease in the threshold for the withdrawal response to a mechanical pressure stimulus applied to the inflamed paw. Both acute inflammation and the associated hyperalgesia resolved within 4 days, and the animal was left with no signs of ongoing inflammation or hyperalgesia. Indeed, carrageenan injection is often used as a model of simple acute inflammatory pain. However, when the paw was challenged with a new inflammatory stimulus, even weeks later, a dramatically enhanced hyperalgesic response was apparent (38). Thus, the injection of a low dose of an inflammatory cytokine, e.g., prostaglandin E_2_ (PGE_2_), which in a naive rat paw would cause only brief hyperalgesia lasting less than 4 h, now evoked hyperalgesia lasting at least 24 h. In addition to this prolongation, hyperalgesic priming also causes an increase in the magnitude of hyperalgesia: the dose-response relationship for PGE_2_-induced hyperalgesia is shifted to the left by more than one order of magnitude (40).

Indomethacin, in the present study, did not modify the chronic NP. Supraspinal and cortical areas participate in the chronic stages of NP (14). Supporting this point of view, Medeiros et al. (11) demonstrated a key role played by the rostral divisions of the frontal cortex in the elaboration of the chronic stages of NP. In fact, the prelimbic division of the medial prefrontal cortex (PrL-mPFC) is critically implicated in the chronic pain phenomenon twenty-one days after the adapted nervus ischiadicus CCI procedure to produce mechanical allodynia. The synaptic function blocker cobalt chloride, when microinjected into the PrL-mPFC cortex, attenuated mechanical allodynia twenty-one and twenty-eight but not seven and fourteen days after the nervus ischiadicus CCI (11).

**Figure 3:**
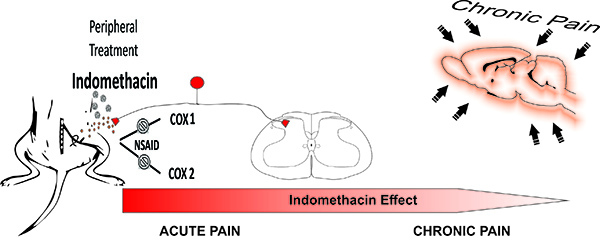
The peripheral treatment with the nonsteroidal anti-inflammatory drug (NSAID) indomethacin attenuates mechanical allodynia during the organization (acute pain) but not the maintenance (chronic pain) of peripheral neuropathic pain induced by nervus ischiadicus chronic constriction injury. Indomethacin did not modify chronic neuropathic pain. Supraspinal and cortical areas participate in the chronic stages of neuropathic pain.

In accordance with Xu and collaborators (13), the pathophysiological mechanisms that underlie the genesis and potentiation of NP have three stages: first, the peripheral nerve suffering the induction of pain, followed by spinal cord neuronal activation, and finally a supraspinal or cortical potentiation. The transmission of noxious stimuli from peripheral receptors to the cerebral cortex involves multiple central ascending pathways. Concerning that, indomethacin has a peripheral action modulating the induction of neuropathic pain, and supraspinal/cortical regions are recruited later during the chronification of NP, which could explain the failure of indomethacin during the later phase of the chronic neuropathic pain phenomenon (Figure 3).

## Supplementary Material

Click here to view [pdf].
